# Development of targeted therapy for bladder cancer mediated by a double promoter plasmid expressing diphtheria toxin under the control of H19 and IGF2-P4 regulatory sequences

**DOI:** 10.1186/1479-5876-8-134

**Published:** 2010-12-16

**Authors:** Doron Amit, Abraham Hochberg

**Affiliations:** 1 The Hebrew University of Jerusalem, Biological Chemistry, Jerusalem 91904, Israel

## Abstract

**Background:**

The human IGF2-P4 and H19 promoters are highly active in a variety of human cancers (including bladder cancer), while existing at a nearly undetectable level in the surrounding normal tissue.

Single promoter vectors expressing diphtheria toxin A-fragment (DTA) under the control regulation of IGF2-P4 or H19 regulatory sequences (IGF2-P4-DTA and H19-DTA) were previously successfully used in cell lines, animal models and recently in human patients with superficial cell carcinoma of the bladder (treated with H19-DTA). However this targeted medicine approach could be limited, as not all cancer patients express high levels of H19. Hence, a double promoter DTA-expressing vector was created, carrying on a single construct two separate genes expressing the diphtheria toxin A-fragment (DTA), from two different regulatory sequences, selected from the cancer-specific promoters H19 and IGF2-P4.

**Methods:**

H19 and IGF2-P4 gene expression was tested in samples of Transitional Cell Carcinoma (TCC) of the bladder by in-situ hybridization (ISH) and by quantitative Real-Time PCR (qRT-PCR). The therapeutic potential of the double promoter toxin vector H19-DTA-IGF2-P4-DTA was tested in TCC cell lines and in heterotopic and orthotopic animal models of bladder cancer.

**Results:**

Nearly 100% of TCC patients highly expressed IGF2-P4 and H19, as determined by ISH and by qRT-PCR. The double promoter vector exhibited superior tumor growth inhibition activity compared to the single promoter expression vectors, in cell lines and in heterotopic and orthotopic bladder tumors.

**Conclusions:**

Our findings show that bladder tumors may be successfully treated by intravesical instillation of the double promoter vector H19-DTA-P4-DTA.

Overall, the double promoter vector exhibited enhanced anti-cancer activity relative to single promoter expression vectors carrying either gene alone.

## Introduction

Bladder cancer is the fourth most commonly diagnosed malignancy in men and the ninth most commonly diagnosed malignancy in women, (NCI annual report 2009).

Urinary bladder neoplasm can be grouped into three different categories: Superficial, invasive and metastatic. At presentation, 75% of the tumors are superficial, 20% are invasive and up to 5% have de novo metastasis. The wall of the bladder is lined with cells called transitional cells. More than 90% of urothelial cancers in the bladder are transitional cell carcinomas (TCC). Other important histologic types include squamous cell carcinoma and adenocarcinoma [1].

At presentation, tumors are usually limited to the bladder mucosa (Ta) or submucosa (T1). These tumors can be removed by transurethral resection (TUR), but tend to recur in 50-70% of the patients. Measures to decrease this high recurrence rate include intravesical chemotherapy and immunotherapy (BCG - Bacillus Calmet-Guerin). These treatments decrease the recurrence rate, but are associated with side effects and frequent failures [1].

The target population of this study is patients with superficial bladder cancer refractory to conventional therapies. Conventional therapies have focused on mass cell killing without specific targeting and often cause damaging and severe side effects to normal tissues. The development of targeted therapeutic strategies based on human cancer gene therapy is an attractive approach.

Based on early studies of our group and others, the transcriptional regulatory sequences of the H19 and IGF2 genes emerged as candidates for cancer targeted therapy. H19 and IGF2 (the human P3 and P4 promoters) are onco-fetal genes and are oncogenes [2-4], expressed in the fetus and in a broad spectrum of tumors, but rarely in normal adult tissues [5-7]. H19 is a paternally-imprinted, oncofetal gene that encodes a RNA (with no protein product) acting as a "riboregulator" [8], which is expressed at substantial levels in embryonic tissues, in different human tumor types, and marginally or not expressed in the corresponding tissues of the adult [6,9]. The 67-aa IGF2 is a member of the insulin like growth factor family that is involved in cell proliferation and differentiation [10]. The human IGF2 gene contains 9 exons (E1-9) and 8 introns [10,11], and is transcribed from 4 different promoters (P1-P4) producing 4 different transcripts [11-13]. All four transcripts share a common coding region and a common 3.9 kb 3-UTR, but variable 5-UTRs [11]. IGF2 is an imprinted gene that is almost exclusively expressed from the paternal allele [14-16]. The P3 and P4 promoters are the major IGF2 promoters during embryogenesis and tumor development, while P1 is exclusively active in adult liver tissue and P2 activity is rarely detected in adult human tissue [10]. Increased expression of IGF2 as a result of the loss of its imprinting is frequently seen in a variety of human tumors [16-18]. In addition, abnormal signal transduction and/or promoter activation was reported as a major mechanism for the IGF2 overexpression in a variety of tumors including bladder carcinoma, hepatocellular carcinoma, breast cancer, ovarian cancer and prostate cancer [19-22]. The human H19 gene lies within 200 kb downstream of the paternally expressed IGF2 gene at 11p.15.5. These two genes are frequently coordinately regulated, both in terms of their common expression pattern and are reciprocal imprinting. Enhancers located downstream of H19 stimulate transcription of both genes [23].

We have shown that IGF2 or H19 are significantly expressed in 50-84% of human bladder carcinomas, respectively [7,24]. Our group has previously reported the construction of single promoter vectors expressing diphtheria toxin A-chain gene, under the control of IGF2-P4 or H19 regulatory sequences (IGF2-P4-DTA and H19-DTA). We showed that these constructs were able to selectively kill tumor cell lines and inhibit tumor growth in vitro and in vivo in accordance to the transcriptional activity of the above-mentioned regulatory sequences [7,25]. Moreover, our group used this therapeutic approach (using H19-DTA) in a successful treatment of a patient suffering from bladder cancer for a period of over 6 years [25], a phase I/IIa clinical trial using this therapeutic approach has been successfully completed [26] and the FDA has approved the initiation of following phase IIb clinical trial. However, there are TCC cells that do not express H19 and as a result, there are patients that could not match this treatment.

Thus for the first time, in the present study, a double promoter DTA-expressing vector was created, carrying on a single construct two separate genes expressing the diphtheria toxin A-fragment (DTA), from two different regulatory sequences, H19 and IGF2-P4 ('H19-DTA-P4-DTA' vector). This novel approach, create a new family of plasmids regulated by two regulatory sequences, which in their natural genome position are both proximately located and are reciprocally imprinted. This is a new biology concept, which mimics the unique biology reciprocity relations phenomenon of IGF2 and H19.

This vector was then used to transfect and to eradicate tumor cells in culture or to inhibit tumor growth (*in vivo*), in heterotopic and orthotopic bladder tumor models.

The activity of the double promoter vector was tested and compared to the activity of the single promoter vectors.

The results showed enhanced activity of the double promoter vector, H19-DTA-P4-DTA, relative to the single promoter expression vectors carrying either DTA sequence alone.

## Materials and methods

### Cell culture

The human bladder carcinoma cell line T24P was obtained from the American Type Culture Collection (ATCC; Rockville, MD). The human bladder carcinoma cell line HT-1376 was kindly provided by Prof W. Schulz, Heinrich-Heine University of Dusseldorf, Germany. Cells were grown to confluency in a humidified incubator with 5% CO2 in polystyrene culture flasks and were maintained in DMEM-F12 (1:1) medium containing 10% fetal calf serum.

### RNA Isolation, cDNA Synthesis and PCR

RNA was extracted from cell lines or frozen tissue blocks, using the RNA STAT-60TM Total RNA/mRNA isolation reagent, according to the manufacture's instructions. The RNA was treated by RNAse-free DNAse I to eliminate any contaminating DNA. Total cDNA was synthesized from 2 μg total RNA in 20 μl reaction volume with 10 ng/μl of the oligo-(dT)15 primer and 10 units/μl M-MLV Reverse Transcriptase according to the manufacturer instructions. 2 μl of cDNA samples were taken for the amplification of the different transcripts using the different primers. The amplification conditions were 95°C for 2 min, followed by 30 cycles of 94°C for 30 sec, 59°C for 45 sec and 72°C for 60 sec, and finally 72°C for 5 min. The PCR reactions were carried out in 25 μl volumes in the presence of 6 ng/μl of each of the forward and the reverse primers using 0.05 units/μl of Taq polymerase according to the kit instructions (Takara). The forward (5'-CCGGCCTTCCTGAACA) and reverse (5'-TTCCGATGGTGTCTTTGATGT) primers designed for the detection of H19 RNA are spanning exons 2-3 and from exon 5 respectively, in order to validate that the PCR product is of the H19 RNA transcript and not from the endogenous H19 gene. The primers designed for the detection of IGF2-P4 RNA were designed to bind at exon 6 (5'-TCCTCCTCCTCCTGCCCCAGCG), for the P4 transcript in the forward direction and the reverse primer (5'- CAGCAATGCAGCACGAGGCGAAGCC) was designed to bind the 3' end of exon 7 and the 5' end of exon 8 without the introns in between. The integrity of the cDNA was assayed by PCR analysis of the ubiquitous, cell cycle independent, histone variant, H3.3 [7]. The PCR products were separated by electrophoresis on 2% gel agarose, and detected by ethidium bromide dye.

### Quantitative Real time PCR (qRT-PCR)

Human TCC samples were obtained from patients undergoing TUR or radical cystectomy at Hadassah Hospital (Hadassah Hebrew University Medical Center, Jerusalem, Israel), following permission of the local IRB.

Samples were analyzed using Mx3000p qRT-PCR detection system and its appropriate software Mx3000p qRT-PCR Software version 3.20 (Stratagene, La. Jolla, CA). Samples contained 10 μl of absolute blue qRT-PCR master mix (ABgene, Epsom, UK), 2 μl of samples, 500 nM of primers and 100 nM of TaqMan MGB probes (Applied Biosystems, Foster City, CA, USA) [27]. Amplification was done by an initial step of enzyme activation at 95°C, followed by 40 cycles of 95°C for 15 sec and 60°C for 1 min. The amount of FAM fluorescence released from each tube was measured as a function of the PCR cycle number. To estimate the sensitivity of the real-time PCR procedure, three separate plasmid DNA controls were used with 10 fold serial dilutions of known quantities. For H19 analysis, starting from 0.2 ng (9 × 10^7 ^copies) up to 0.2 × 10^-7^ng (≤ 9 copies of plasmid DNA) were used. For IGF2-P4 analysis, starting from 0.2 ng (3 × 10^7 ^copies) up to 0.2 × 10^-7^ng (≤ 3 copies of plasmid DNA) were used. Simultaneous amplifications of standard dilution series were then performed. The number of target copies was determined using the standard curve created in the same run. The qRT-PCR assays were accepted when a positive signal was detected in all positive control dilutions and no signal was detected in the negative sample controls. The threshold for high expression level was set as >10,000 DNA copies number (per 0.2 μg c-DNA). These experiments were performed in triplicates.

### DIG-labeled Probe Synthesis

A PCR strategy was used to generate template DNA for synthesis of labeled RNA probes.

Forward primers for the human H19 and IGF2-P4 genes were designed. Each primer contain Sp6 promoter sequence in its 5'-end. Accordingly, a reverse primer was also designed with T7 promoter sequence incorporated in its 5'-end. The PCR products obtained for the H19 and IGF2-P4 transcript were purified from the gel by the DNA and Gel Band Purification Kit (Amersham), and used as templates for the PCR-based incorporation of T7 and Sp6 RNA polymerase promoter. The PCR conditions used to generate the T7/Sp6 templates were the same as described earlier for the synthesis of H19 and IGF2 specific transcripts. The PCR products (containing T7 and Sp6 promoters) were purified from the gel, sequenced and found to be identical to the relevant published sequences in the gene bank. 100 to 200 ng from the purified products were used as templates for the T7 and Sp6 polymerase (2 units/μl), according to the manufacturer instructions in the presence of 2 units/μl RNase inhibitor. T7 and Sp6 promoters were respectively used to drive the synthesis of the antisense and the control sense Digoxigenin-labeled UTP probes. The resulting probes were treated by 2 units of RNase free DNase I, pelleted and resuspended in appropriate volume of DEPC-treated double distilled water. The sizes of the synthesized probes were analyzed by running on 4% denaturing agarose minigel, and their labeling efficiency was determined by dot blot analysis.

### In situ hybridization (ISH)

The non radioactive ISH washing and treatments were as described in [7]. Each section was rehydrated by 30 μl of the hybridization solution containing about 30 ng of DIG labeled RNA probe at 52°C. The ISH was performed on successive slides of TCC tissue for H19 and IGF2-P4 transcripts. The intensity of hybridization signal was indicated as (0) for no staining, (+1) for weak, (+2) for moderate and (+3) for strong signals. The distribution of the hybridization signal was referred to as up to one third of the cells, + (1), one to two thirds, ++ (2), and more than two thirds, +++ (3). Therefore the total scoring (intensity + quantity) for each sample varied from 0 (no expression) to 6 (very high expression). Low expression was set as total scoring of 0 < X < 3 and high expression was set as total scoring of 3 ≤ X ≤ 6.

### Plasmid construction

**Figure 1:**
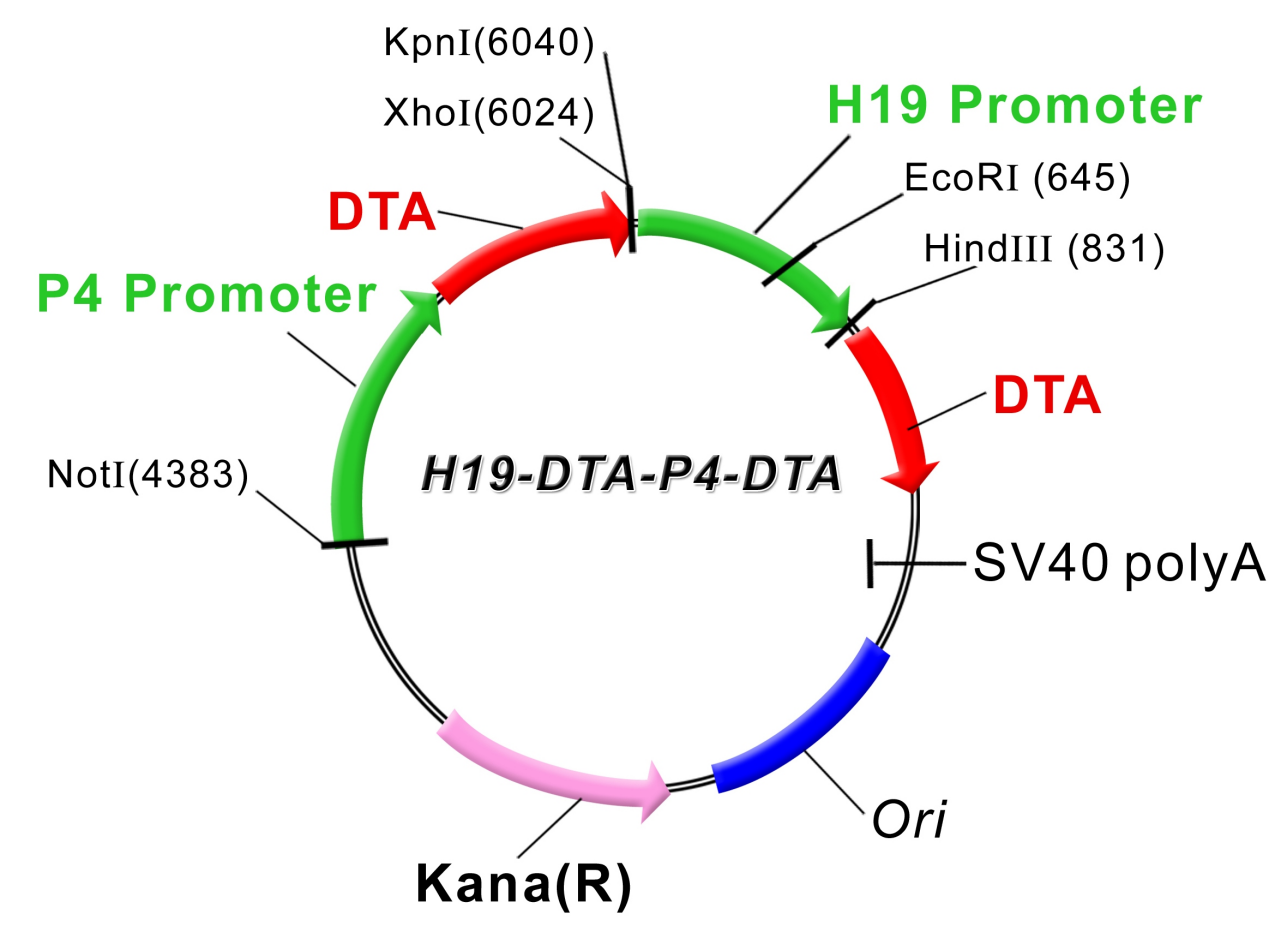
**A schematic illustration depicting the construction of the double promoter H19-DTA-P4-DTA expression vector**: The coding sequence of each DTA is under the transcriptional control of both H19 and IGF2-P4 promoter sequences, respectively, Kana (R) - kanamycine resistance gene.

The H19-Luc plasmid which contains the luciferase gene under the control of the human H19 promoter region from nucleotide -818 to + 14 was prepared as described [28]. The H19-Luc plasmid was digested with XbaI and NcoI, and the insert of the luciferase gene (luc) was replaced by the Diphtheria toxin A chain (DTA) coding region to yield the H19-DTA construct. The DTA gene was prepared from the pIBI30-DT-A plasmid (kindly donated by Dr. Ian Maxwell, University of Colorado, USA). The human IGF2-P4 promoter from the Hup4 vector (described in [11]) (a kind gift from Prof. P.E. Holthuizen, University of Utrecht, The Netherlands) were constructed by GENEART into the pGL3 basic vector (Luc-1) (Promega, Madison, MI), which lacks any eukaryotic promoter and enhancer sequences and carries the Kanamycine resistance gene (insert 812 bp), using BstEII and Hind III restriction sites, resulting in the expression vector P4-Luc. The DTA containing vector P4-DTA was designed by replacing the luciferase gene in P4-Luc with the DTA gene between the XbaI and NcoI restriction sites. Each of the cloned promoters and the DTA gene were sequenced and compared to the published sequences of the gene bank. We constructed double promoter expression plasmids, carrying on a single construct two separate genes expressing the diphtheria toxin, from two different regulatory sequences, as follows: H19 + IGF2-P4 promoters (hereinafter "H19-DTA-P4-DTA"; depicted in Figure 1).

A double promoter control constructs was created, using the same strategy, expressing the luciferase reporter gene ('H19-Luc-P4-Luc'). The double promoter expression plasmids were cloned by GENEART™, (Germany)

### Transfection

#### Cationic polymer (jetPEI) transient transfection

The *in vitro *jetPEI™transfection reagent compact the DNA into positively charged particles capable of interacting with anionic proteoglycans at the cell surface and entering cells by endocytosis. The transfection procedure was done as recommended by the manufacturer (Polyplus-transfection, France). A total of 0.1 × 10^6 ^cells/well were grown overnight in a twelve-well Nunc multidish (75 mm). For each well, 2 μg DNA and 4 μl of the jetPEI (N/P = 5) were diluted separately with 50 μl of 150 mM NaCl each, and vortex-mixed gently. The jetPEI solution was added at once to the DNA solution, the mixture was vortex-mixed for 10 seconds and the mixture was incubated for 15 minuets at room temperature. The 100 μl jetPEI/DNA mixture was then applied drop-wise onto the serum containing medium of each well. The transfection experiment was stopped after 48 hours.

#### Luciferase activity

The cells were harvested and the luciferase activity was determined using the luciferase Assay System kit (Promega). The light output was measured using a Lumac Biocounter apparatus. The total protein content of the lysates was determined by the Bio-Rad protein assay reagent and the results were normalized to the total protein and expressed as Light units/μg protein. LucSV40 (Luc-4) was used as a positive control for the efficiency of transfection as it contains the SV40 promoter and enhancer, while Luc-1 that lacks any regulatory sequences was used as a negative control to determine the basal nonspecific luciferase expression, which was found to be negligible in all of the cell lines. All experiments were done in triplicates and the results expressed as mean and standard error.

### In vitro targeted therapy

The cells were cotransfected with 2 μg of the LucSV40 control vector and with the indicated amounts of the DTA expressing vector (H19-DTA, P4-DTA or the DTA double promoter expressing vector H19-DTA-P4-DTA). The same cells were additionally transfected with 2 μg LucSV40 alone in the same experiment. The H19-DTA, P4-DTA and H19-DTA-P4-DTA cytotoxic activity was determined by calculating the % of decrease in the cotransfected LucSV40 activity compared to that of LucSV40 transfected alone in the same cell type. The total protein content of the lysates was determined by the Bio-Rad protein assay reagent and the results were normalized to the total protein and expressed as Light units/μg protein. Therefore the reduction in luciferase activity, reflect the inhibition of protein synthesis activity by the DTA.

### In vivo targeted therapy animal models

All surgical procedures and the care given to the animals were approved by the local committee for animal welfare. Animals were kept in the Hebrew University's animal facility with water and food *ad librum *(all experimental research on animals follow internationally recognized guidelines). The histopathological examinations of the different tumors were performed in consultation with a trained pathologist.

#### Heterotopic nude mice model

Confluent T24P and HT-1376 human bladder carcinoma cells were trypsinized to a single cell suspension and resuspended in PBS. 2 × 10^6 ^T24P cells or HT-1376 cells (in 150 μl volume) were subcutaneously injected into the back of female CD1 nude mice, 6-8 weeks old. 10 days after cell inoculation the developing tumors were measured in two dimensions and randomized to different treatments. Animals were separated to different groups of the same size (n = 6). The ability to inhibit tumor growth by the single promoter DTA expression vectors (P4-DTA, H19-DTA) and by the double promoter DTA expression vector (H19-DTA-P4-DTA) was tested. Intratumoral injections of 25 μg of either DTA expressing constructs (treatment groups) or Luc expressing constructs (control groups) were given 10, 12 and 14 days after cells inoculation. *In vivo *Jet-PEI a 22 kDa linear form of polyethylenimine (PEI) was used as a transfection enhancer reagent. PEI/DNA complexes with a ratio of PEI nitrogen to DNA phosphate of 6 were prepared in a solution of 5% w/v glucose according to the manufacture's instructions. Tumor dimensions were measured, and the tumor volume was calculated according to the formula width^2 ^× length × 0.5. The animals were sacrificed 3 days after the last treatment, the tumors were excised and their ex-vivo weight and volume were measured. Samples of the tumors were fixed in 4% buffered formaldehyde and processed for histological examination for evidence of necrosis and persistent tumor. Computerized measurements of tumor surface area and of the necrotic surface area were made using the Image Pro Plus software (Media cybernetics, Silver Springs, USA).

#### Orthotopic bladder cancer model

Female CD1 nude mice, 6-8 weeks old were used to develop orthotopic superficial bladder tumors. Mice were anesthetized with intra-peritoneal injection of ketamine (85 mg/kg) and xylazine (3 mg/kg). The bladder was catheterized with a 24 gauge catheter, than drained and its mucosa was mildly disrupted with 0.1 ml HCl 0.1N for 15-sec. (The bladder is rather resistant to implantation of cells, and therefore it is necessary to create abrasions in the bladder mucosa of the anesthetized rodent either by acid, in order to increase "tumor take" [29]). The acid was immediately neutralized with 0.1 ml NaOH 0.1N, and the bladder was washed three times with 0.1 ml PBS. Subsequently, a 0.1 ml suspension of PBS containing 10 × 10^6 ^T24P human bladder carcinoma cells was instilled into the bladder. The urethra was ligated with 6/0 silk suture to assure that cells were retained in the bladder. After 2 hours the sutures were removed and the bladders were evacuated by spontaneous voiding. Four healthy mice were left without T24P cells instillation. Seven days after cell instillation, the animals were anesthetized and the bladders were catheterized the same way. The bladders were washed three times with 0.1 ml of PBS. Animals were separated to different groups of the same size (n = 6). Mice of the DTA treatment groups received 20 μg of the toxin vector H19-DTA-P4-DTA. The control group received 20 μg of the reporter vector H19-Luc-P4-Luc. A group of 4 mice were kept with no treatment. The same treatments were repeated after 3 days. The *in vivo*-jetPEI™ was used as a transfection enhancer agent. For preparation of the solution, 2.4 μl of the jetPEI (N/P ratio = 6) in 50 μl glucose 5% (w/v) were mixed with 20 μg of treatment plasmids respectively, in 50 μl of 5% glucose solution. The resulting mixture was vortex-mixed and left for 10-15 minutes at room temperature and subsequently instilled into the mice bladder transurethrally using the catheter as described above. The animals were sacrificed 4 days after the last plasmid instillation, their bladders were removed and the serosal surface and the adjacent sex glands were dissected carefully. Samples of the tumors were fixed in 4% buffered formaldehyde and processed for histological examination for evidence of necrosis and persistent tumor. Computerized measurements of tumor surface area and of the necrotic surface area were made using Image Pro Plus software (Media cybernetics, Silver Springs, USA). Other samples were frozen by liquid nitrogen and stored at -80°C to be analyzed by RT-PCR for evidence of IGF2, H19, luciferase and DTA mRNA expression.

## Results

### Expression of IGF2-P4 and H19 transcripts in human bladder carcinoma tissues determined by ISH or by RT-PCR

The human IGF2-P4 and H19 regulatory sequences are highly active in a variety of human cancers. In this study we present an approach for targeted therapy of bladder carcinoma by driving the DTA expression under the control of IGF2-P4 and H19 regulatory sequences. To evaluate the possible use of IGF2-P4 and H19 regulatory sequences for targeted therapy of bladder cancer, we determined the expression of IGF2-P4 and H19 transcripts by RT-PCR, qRT-PCR and ISH. Human TCC samples were obtained from patients undergoing TUR or radical cystectomy at Hadassah Hospital, following permission of the local IRB.

**Figure 2:**
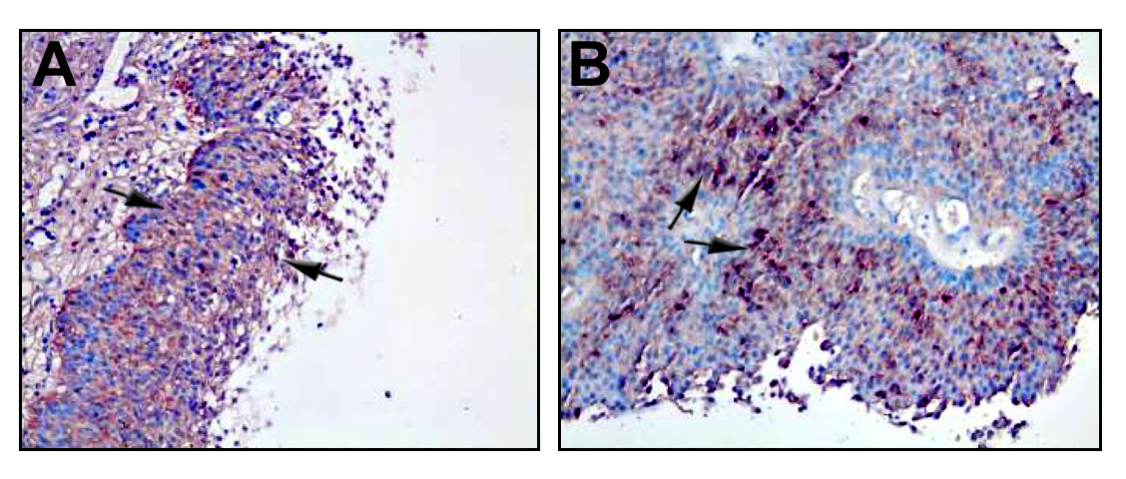
**ISH detection of the expression of IGF2-P4 and H19 transcripts in human TCC tissue samples**: IGF2-P4 (A) and H19 (B) specific transcripts, detected by ISH. The positive stained cells are marked by black arrows (Magnification are ×20).

The samples were first tested for H19 and IGF2-P4 overall expression by RT-PCR or by ISH (Table 1). 38 out of 39 TCC samples examined by RT-PCR showed positive IGF2-P4 transcripts expression and 37 out of 39 TCC samples showed positive H19 expression. Accordingly, 24 out of the 28 TCC samples examined by ISH showed positive IGF2 expression from IGF2-P4 (Figure 2A), and 27 out of the 28 TCC samples showed positive H19 expression (Figure 2B) (Table 1). Taken together the PCR and ISH results show that 62 out of 67 (92.5%) and 64 out of 67 (95.5%) positively expressed varying levels of IGF2-P4 and H19, respectively.

**Table 1:** The H19 and IGF2-P4 overall expression in TCC tissue samples determined by RT-PCR (n = 39) and by in situ hybridization (ISH) (n = 28)

	RT-PCR	ISH	Total
**IGF2-P4**	38/39	24/28	**62/67**

**H19**	37/39	27/28	**64/67**

### Comparison of the expression levels of IGF2-P4 and H19 transcripts in human TCC samples detected by ISH and by qRT-PCR

qRT-PCR and ISH techniques were applied to determine and quantity the level of H19 and IGF2-P4 in human TCC samples.

Human TCC samples (n = 29) were examined by qRT-PCR and the expression level of H19 and IGF2-P4 specific transcripts was determined for each sample by the total number of DNA copies (per 0.2 μg c-DNA). Table 2 demonstrates that high levels of IGF2-P4 and H19 transcripts were found in 83% (24/29) and in 90% (26/29) of the tumor samples, respectively. However the total combined expression of both IGF2-P4 and H19 transcripts, were detected at high expression levels in 100% (29/29) of the tumor samples.

**Table 2:** The expression levels of H19 and IGF2-P4 transcripts in human TCC samples (n = 29), determined by qRT-PCR.

	H19	IGF2-P4	H19 + IGF2-P4
**Low expression**	3/29	5/29	0/29

**High expression**	26/29	24/29	**29/29**

* (High expression: >10,000 DNA copy numbers (per 0.2 μg c-DNA), as described in the "Material and Methods").

Additional human TCC samples (n = 28) were examined by ISH and the expression levels of IGF2-P4 and H19 transcripts were determined by the intensity of the hybridization signal and by the quantity of the stained cells. Table 3 shows that out of 28 TCC samples, high expression levels of H19 and IGF2-P4 were found in 75% (21/28) and 50% (14/28) of the TCC samples, respectively. However when the overall combined expression analysis of the intensity and quantity of both transcripts H19 + IGF2-P4 was determined, then 100% (28/28) of the samples showed positive expression and 26 out of 28 TCC samples (96%) showed high expression levels.

**Table 3:** The endogenous H19 and IGF2-P4 expression levels in TCC tissue samples determined by ISH.

	H19	IGF2-P4	H19 + IGF2-P4
**Low expression**	6/28	10/28	2/28

**High expression**	21/28	14/28	**26/28**

The table shows the level of IGF2-P4 and H19 transcripts, defined as 'Low' or 'High' expression. A semi quantitative scoring system was established to define the levels of H19 expression after ISH (see "Material and Methods").

### Expressing DTA from two different regulatory sequences, using a 'double promoter strategy'

As described, high levels of H19 and IGF2-P4 transcripts were detected in TCC samples. Furthermore, enhanced expression was clearly exhibited for a combined expression of both transcripts (H19 + IGF2-P4).

Therefore, we decided to further investigate the combination use of H19 and IGF2-P4 regulatory sequences for driving toxin gene expression. A double promoter expression vector was created, carrying on a single construct two separate genes expressing the diphtheria toxin A (DTA), from two different regulatory sequences, H19 and IGF2-P4 promoters ("H19-DTA-P4-DTA"; depicted in Figure 1).

### In vitro DTA expression by a single construct containing DTA genes separately expressed from H19 and IGF2-P4 regulatory sequences

**Figure 3:**
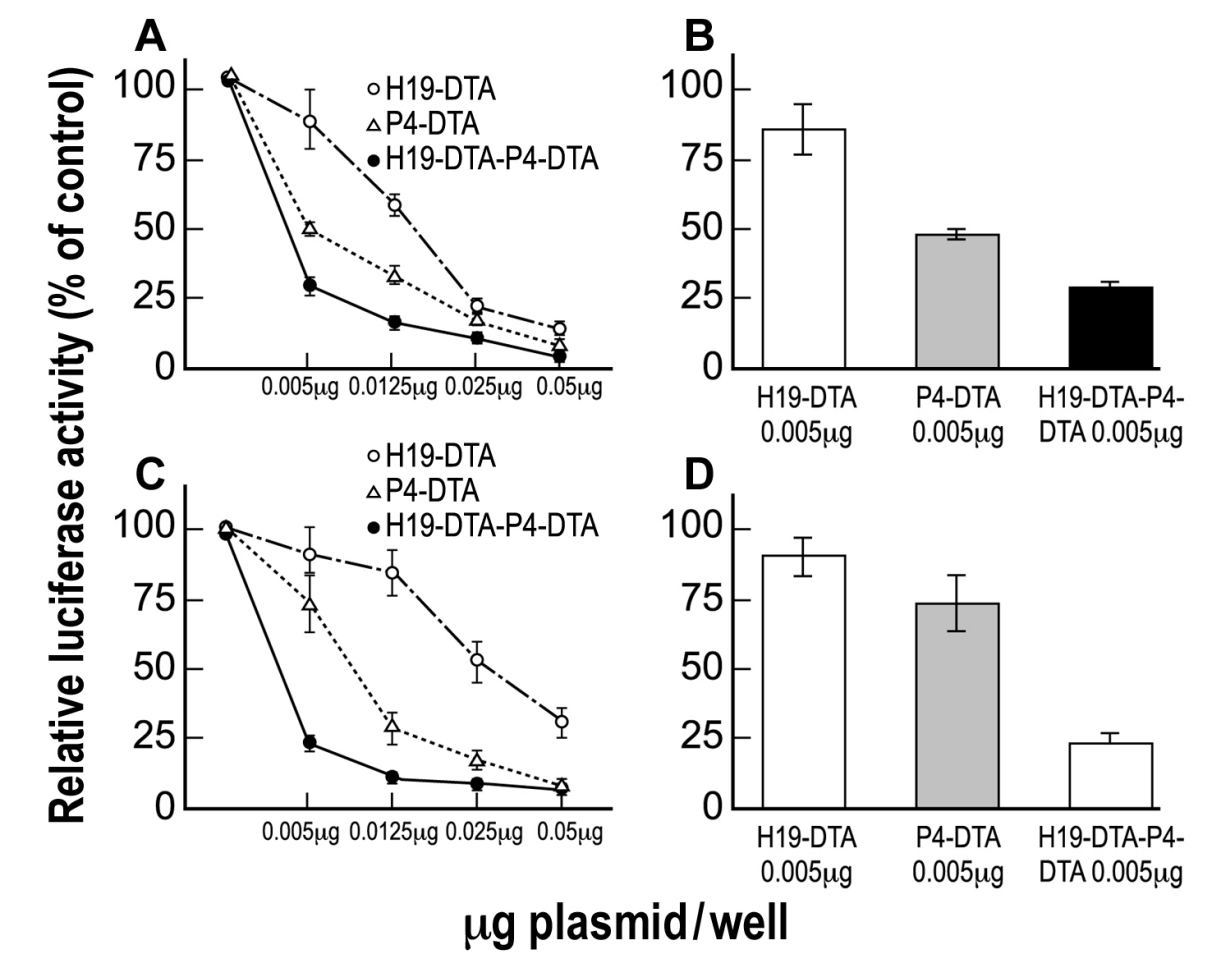
**In vitro enhanced protein synthesis inhibition activity of H19-DTA-P4-DTA in human bladder carcinoma cell lines**: Tumor growth inhibition activity of the H19-DTA, P4-DTA and H19-DTA-P4-DTA vectors in T24P (A-B) and HT-1376 (C-D) cells was measured as a reduction of LucSV40 activity. Cells were cotransfected with 2 μg of LucSV40 and the indicated concentrations of the DTA expressing vectors, or with LucSV40 alone. Transfection experiments were stopped after 48 hours and luciferase activity was assessed. The decrease in LucSV40 activity was determined by comparison to the same cell type transfected with LucSV40 alone as a measure for cytotoxicity. The diverse effect of each vector at the lowest plasmid transfected concentration is indicated (B, D).

The activity of the double promoter construct H19-DTA-P4-DTA was first tested *in vitro *by determining its ability to lyse two different human bladder carcinoma cell lines, relative to the single promoter constructs. Anti-tumor therapeutic activity was determined by measuring the inhibition of luciferase activity following co-transfection with LucSV40. T24P and HT-1376 TCC cells were co-transfected with the indicated vectors (H19-DTA, P4-DTA, or H19-DTA-P4-DTA) in a dose-response manner at the indicated concentrations (Figure 3) and with 2 μg of LucSV40. Luciferase activity as an indicator of survival of the transfected cells was determined and compared to that of cells transfected with LucSV40 alone. H19-DTA or P4-DTA was able to drive the expression of the DTA gene and thus reduce luciferase activity in a dose-response manner. H19-DTA-P4-DTA, however, exhibited far enhanced efficiency in lysing the cancer cell lines, relative to each of the single promoter constructs, in T24P cells (Figure 3A-B) and in HT-1376 cells (Figure 3C-D). The double promoter expressing vector H19-DTA-P4-DTA was able to reduce the LucSV40 activity to more than 70% at concentrations as low as (0.005 μg/well) in T24P (Figure 3B) and HT-1376 (Figure 3D) cells, respectively. Less significant inhibition was obtained by H19-DTA or P4-DTA at the same concentrations (0.005 μg/well) in T24P (Figure 3B) and HT-1376 (Figure 3D) cells.

### In vivo tumor growth inhibition by the double promoter vector in bladder cancer animal models

We used the double promoter construct, H19-DTA-P4-DTA assessing its tumor growth inhibition activity, by DTA expression *in vivo *using heterotopic and orthotopic animal models for bladder cancer.

### Expression of IGF2-P4 and H19 transcripts in heterotopic subcutaneous tumors

**Figure 4:**
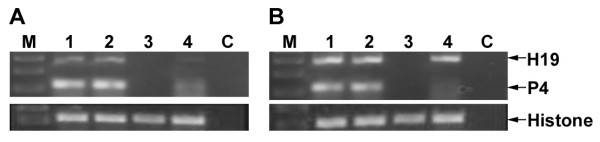
**The expression of H19 and IGF2-P4 in heterotopic subcutaneous tumors determined by RT-PCR**: The expression of H19 and IGF2-P4 transcripts in heterotopic subcutaneous tumors after injection of T24P (A) or HT-1376 cells (B) was determined by RT-PCR. "M": 100-bp molecular weight marker, lanes 1-2: heterotopic subcutaneous tumors from different mice induced by injection of T24P (A) or HT-1376(B) cells, lane 3: subcutaneous tissue of normal mouse, lanes 4: T24P (A) or HT-1376(B) cell lines, "C": negative control for PCR. The sizes of the PCR products are 300 bp for human H19, 119 bp for IGF2-P4 and 213 bp for Histone 3.3 internal control, respectively.

In order to develop a model for heterotopic bladder tumors, T24P or HT-1376 human bladder cancer cells were subcutaneously injected into the dorsa of 6-7 weeks old CD-1 (nude) female mice. Tumors were developed 10 days after cell injection, dissected and total RNA was extracted from the tumors. The expression of IGF2-P4 and H19 RNA was determined by RT-PCR analysis. High expression of IGF2-P4 and H19 RNA was found in the heterotopic tumors induced by T24P cells (Figure 4A lanes 1-2) or by HT-1376 cells (Figure 4B lanes 1-2). Moreover there was no H19 and IGF2 expression in normal control mice (lane 3). Interestingly, the expression of H19 and IGF2-P4 RNA in the heterotopic tumors was higher compared to the *in vitro *expression of T24P cells (lane A4) or HT-1376 cells (lane B4) used for inoculation.

### Tumor growth inhibition by the double promoter vector in heterotopic bladder carcinoma model

**Figure 5:**
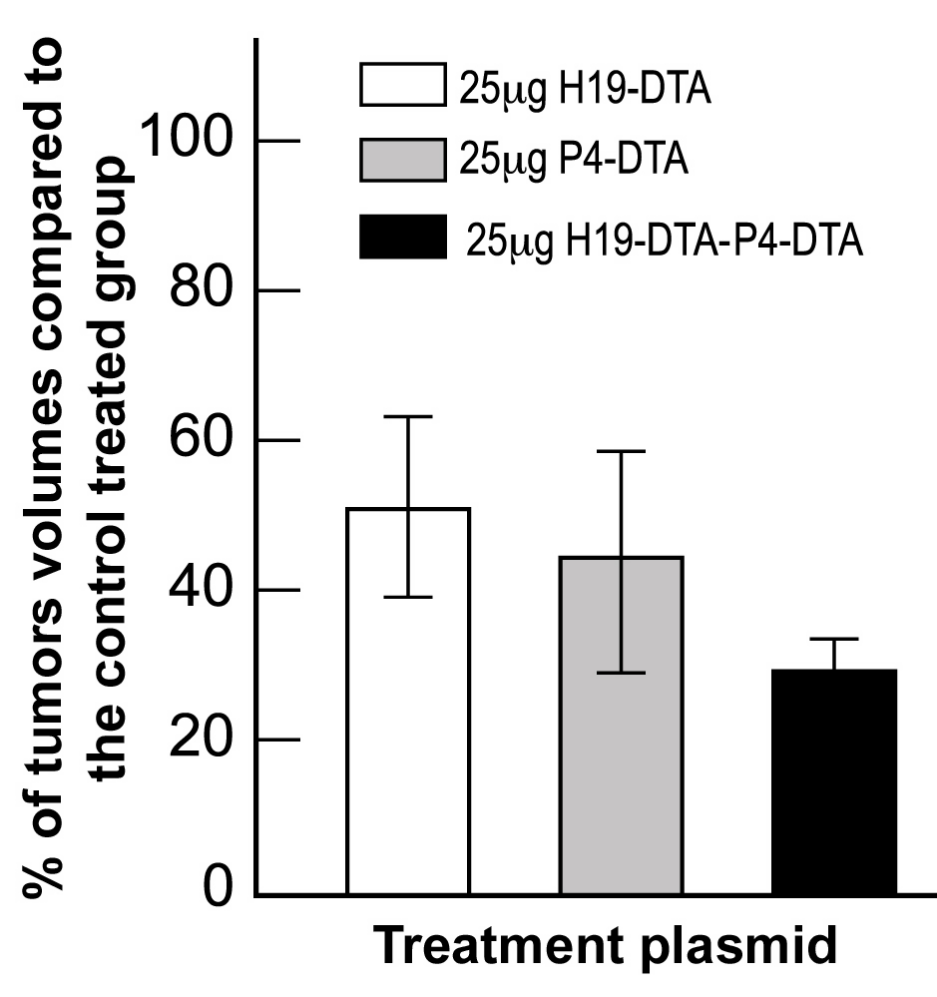
**In vivo inhibition of heterotopic tumors in response to H19-DTA-P4-DTA treatments**. Inhibition of tumor growth in response to H19-DTA, P4-DTA, or H19-DTA-P4-DTA treatments is shown. The tumor sizes of tumors treated with the DTA expressing vector, or with control luciferase expressing vectors were determined prior to each treatment and before sacrifice. The fold increase in tumor volume was calculated relative to the initial volume at the day of the first treatment.

The tumor growth inhibition activity of H19-DTA-P4-DTA was tested in heterotopic bladder tumors, induced by T24P cells. T24P cells were subcutaneously injected into the back of 6-7 weeks old CD-1 female mice in order to develop a model for heterotopic bladder cancer. 10 days after subcutaneous cell inoculation, the mice developed measurable heterotopic tumors for testing. The therapeutic potency of the vectors was tested by direct intratumoral injection of 25 μg of the DTA expression vectors (H19-DTA, P4-DTA, or H19-DTA-P4-DTA), or of the control vectors (H19-Luc, P4-Luc, or H19-Luc-P4-Luc) into each heterotopic bladder tumor. Tumors sizes were determined and the *in vivo *fold increase of the tumor size was calculated prior to each treatment and before sacrifice. Three injections of H19-DTA or P4-DTA (Figure 5) at two-day intervals were able to inhibit tumor development by 49% (P = 0.001) and 55.5% (P = 0.005), respectively compared to H19-Luc and P4-Luc treatments. However, three injections of the double promoter plasmid H19-DTA-P4-DTA at two-day intervals inhibited tumor development by 70% (P < 0.001) compared to H19-Luc-P4-Luc treatment (Figure 5). The double promoter construct thus exhibited enhanced ability to inhibit tumor development *in vivo*, compared to each of the single-promoter constructs (H19-DTA, or P4-DTA).

**Figure 6:**
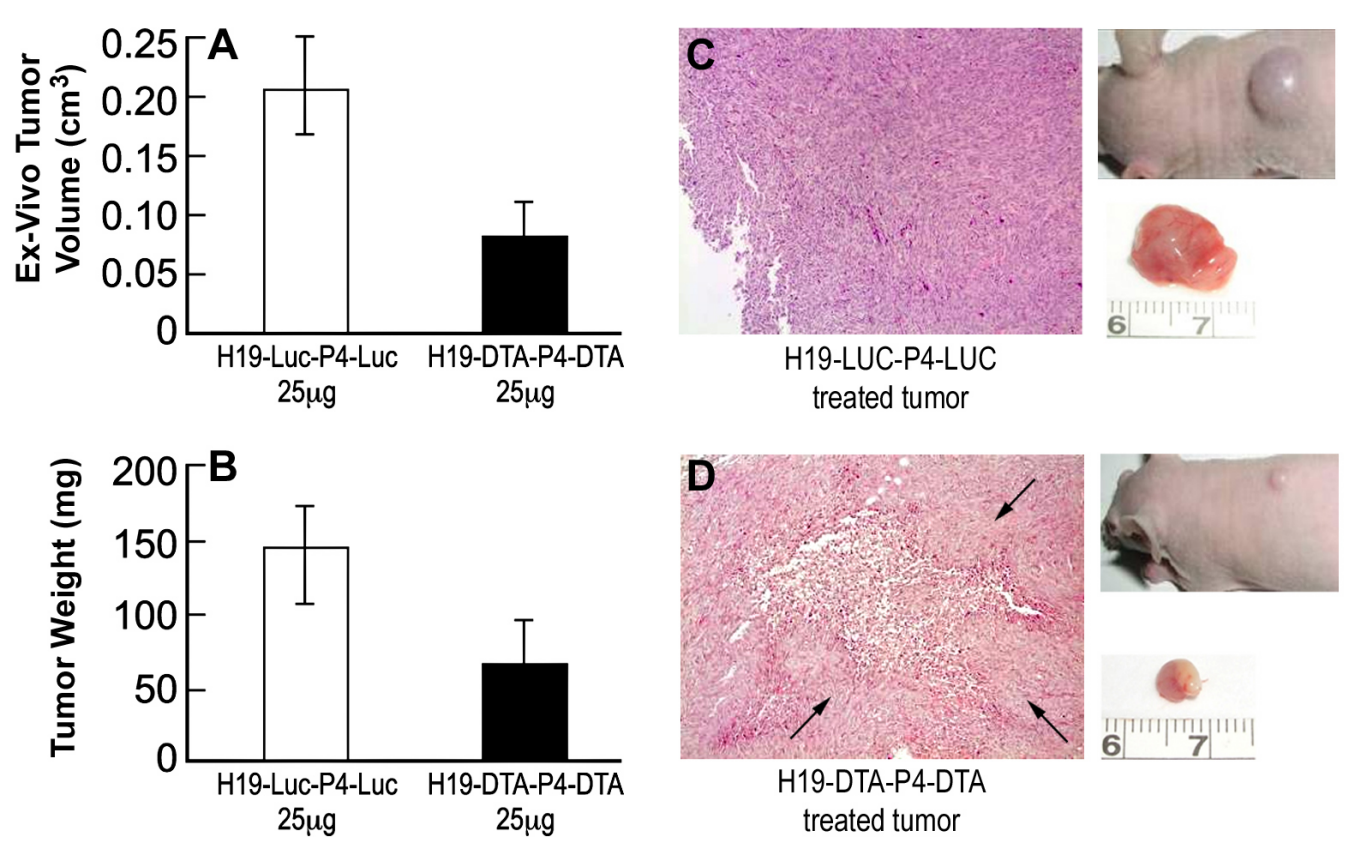
**Heterotopic tumors treated by H19-DTA-P4-DTA**. Heterotopic bladder tumors treated with H19-DTA-P4-DTA vector (black) or with H19-Luc-P4-Luc control vector (white) were excised and the ex-vivo tumors volume were measured (A) and weighted (B). C-D: Necrosis of heterotopic tumors treated with H19-DTA-P4-DTA: Hematoxylin Eosin (HE) staining (×10) of representative sections of tumors treated with H19-Luc-P4-Luc (C), or with H19-DTA-P4-DTA (D). The necrotic areas are indicated by arrows (D). Inserts are macroscopic photographs of the heterotopic tumors.

To confirm the difference between the H19-DTA-P4-DTA and H19-Luc-P4-Luc groups, tumors were excised and their *ex vivo *volume and weight were determined as well. Mice treated with H19-DTA-P4-DTA exhibited a 61% (P < 0.001) reduction of the *ex-vivo *tumor volume (Figure 6A) and a 54% (P = 0.002) reduction of the *ex-vivo *tumor weight (Figure 6B) compared to H19-Luc-P4-Luc treated mice. The consistency of the results, in measurements of the *ex-vivo *tumors as well, eliminates any unrelated difference of the measurements (such as subcutaneous inflammation swelling due the necrosis reaction, etc.).

### In vivo tumor growth inhibition of orthotopic bladder tumors by the double promoter vector

Transurethral implantation of human bladder cancer cells into the mouse bladder (orthotopic model) provides a more relevant tool for the investigation of the biology and therapy of bladder cancer than subcutaneous implantation of bladder cancer cells (heterotopic model). Therefore, a mouse model was developed by intravesical instillation of T24P human bladder carcinoma cells onto the wall of the mouse bladder *in vivo*. This model was then used for testing the tumor growth inhibition activity of the double promoter H19-DTA-P4-DTA vector.

### Treatment of the orthotopic tumors

**Figure 7:**
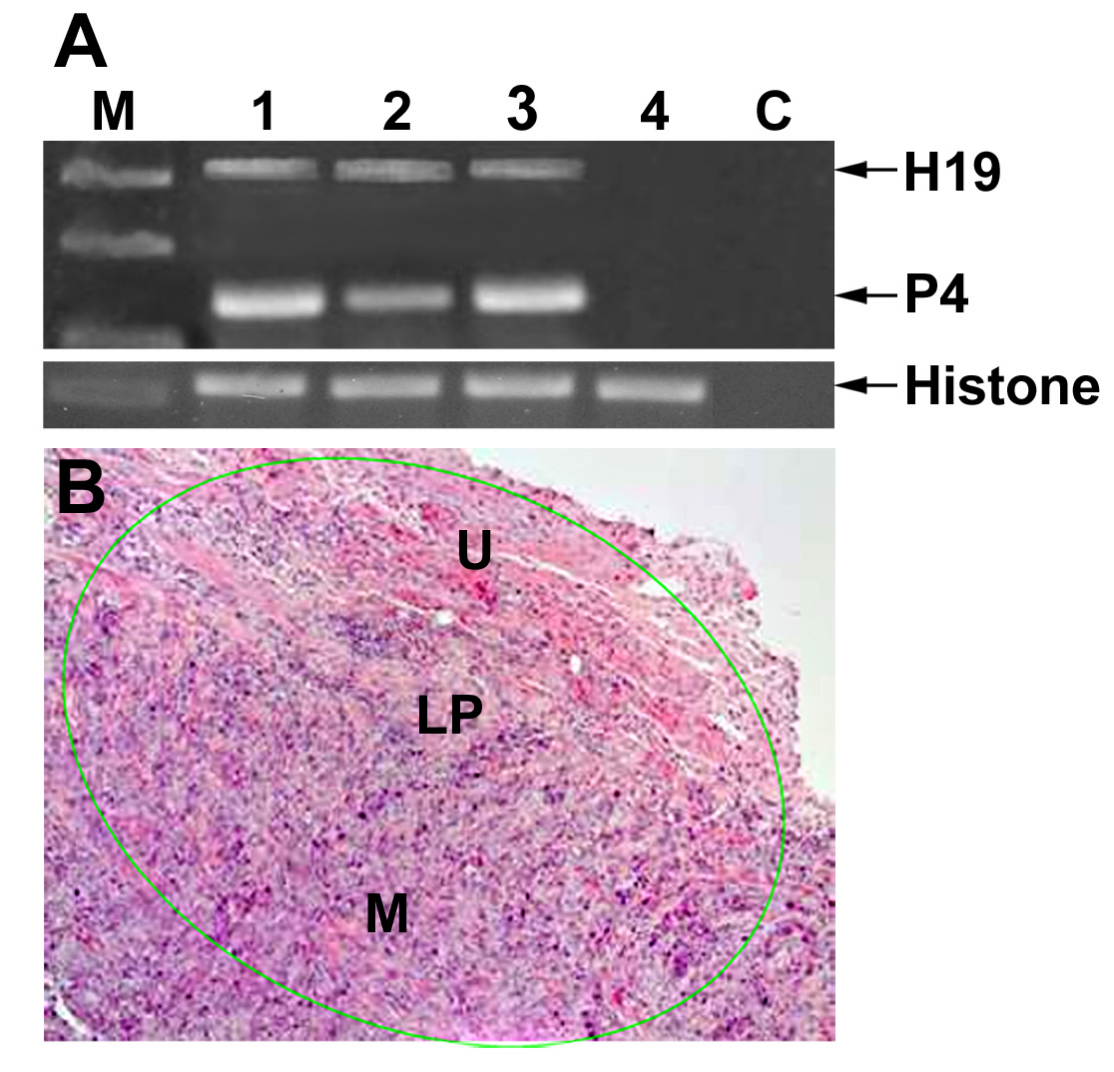
**Orthotopic bladder tumors kinetics, 14 days after intravesical cells instillation**: **A)**. "M": 100-bp molecular weight marker, lanes 1-3: orthotopic bladder tumors from different mice induced by intravesical instillation of 10 × 10^6 ^T24P cells, lane 4: bladder of normal mouse, "c": negative control for PCR. **B)**. HE staining (×10) of a representative section of orthotopic bladder (14 days after intravesical inoculation of 10 × 10^6 ^T24P cells). The tumor area is indicated (by green line). ('U', urothelium, 'LP', lamina propria, 'M', muscularis).

Considerably large tumors were obtained 14 days after the T24P cells inoculation. As shown in Figure 7A high expression of both H19 and IGF2-P4 was determined by RT-PCR, in orthotopic bladder tumors, sacrificed 14 days after cells inoculation. By this time the tumors already started to invade the lamina propria as well as the superficial and deep muscle (Figure 7B). These tumors would not therefore be suitable to start the treatment by the DTA therapeutic constructs because it does not resemble the stage at which most of the cases in human (more than 75%) consult the physician. Therefore, the treatment was started 7 days after cells inoculation, which was enough to develop smaller and less invasive orthotopic tumors than after 14 days. The treatment group (n = 6) was intravesically treated with 20 μg of H19-DTA-P4-DTA and the control group (n = 6) received 20 μg of H19-Luc-P4-Luc. Three days later the same treatments were repeated. Additional four control healthy mice were intravesically treated with HCL/NaOH at the beginning of the experiment with no additional following treatments. The animals were sacrificed at the end of the experiment (4 days after the second treatment), their bladders were processed for assessment of tumor sizes and for PCR and histology analyses (see Materials and Methods).

**Figure 8:**
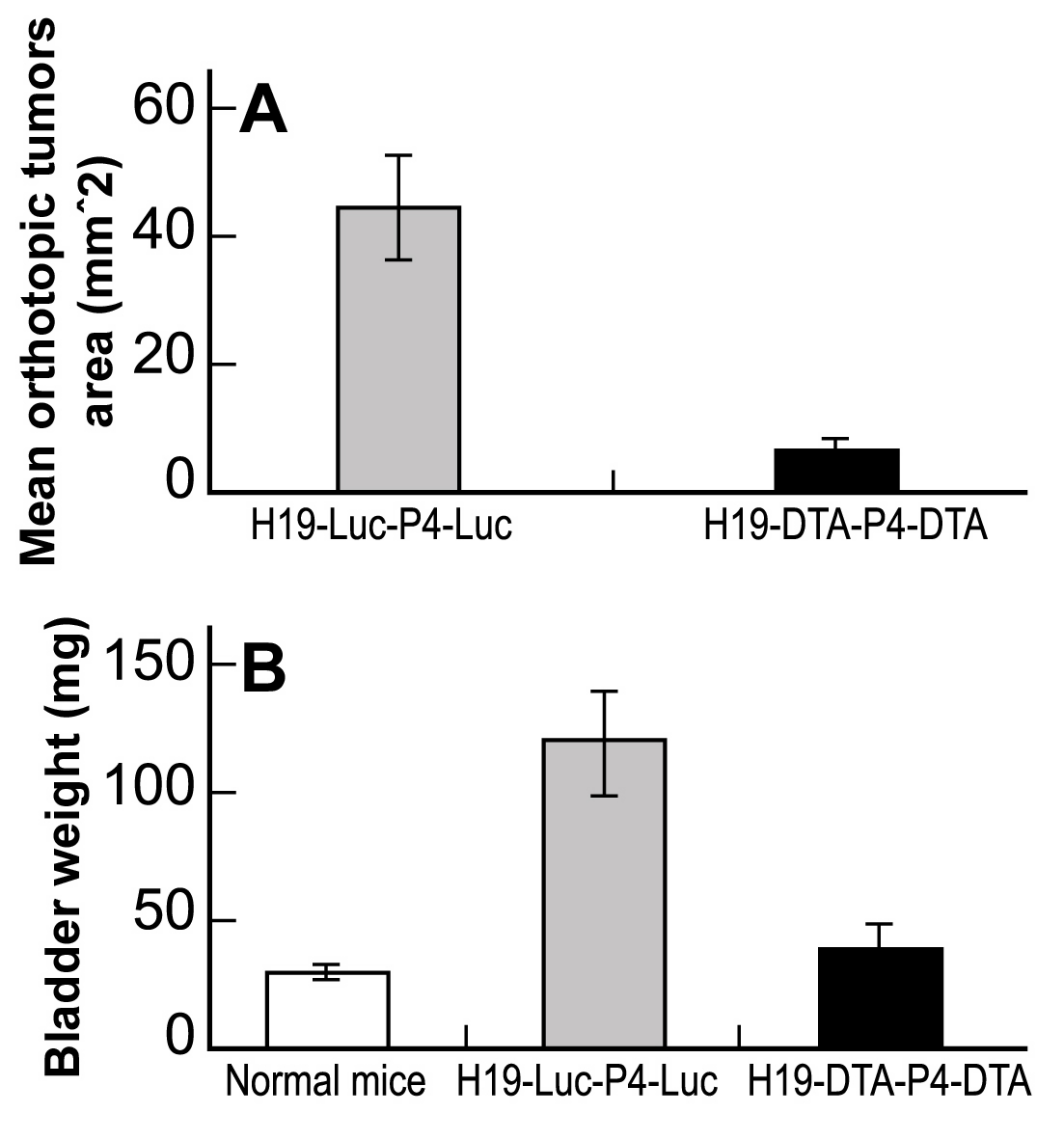
**The effect of intravesical treatment with H19-DTA-P4-DTA vector in orthotopic bladder carcinoma**: Orthotopic tumors were induced by intravesical instillation of T24P cells, in nude mice bladders. 7 days later, mice of each group (n = 6) received an intravesical treatment with 20 μg of H19-DTA-P4-DTA, or H19-Luc-P4-Luc for each mouse. The same treatments were repeated after 3 days, and 4 days later mice were sacrificed. The bladders of both groups were excised, weighted, and the area of the malignant tissue of each bladder was determined by ImagePro Plus software. Another 4 healthy mice were used as control. The total tumor area of each bladder was determined and the mean of the total areas was calculated for each group. The Mean and SD of bladder tumor area (A) and weight (B) are shown.

As can be seen in Figure 8, two treatments of 20 μg of H19-DTA-P4-DTA in three day intervals were able to inhibit tumor growth significantly as reflected by measuring the size of the tumors and by bladders weight.

**Figure 9:**
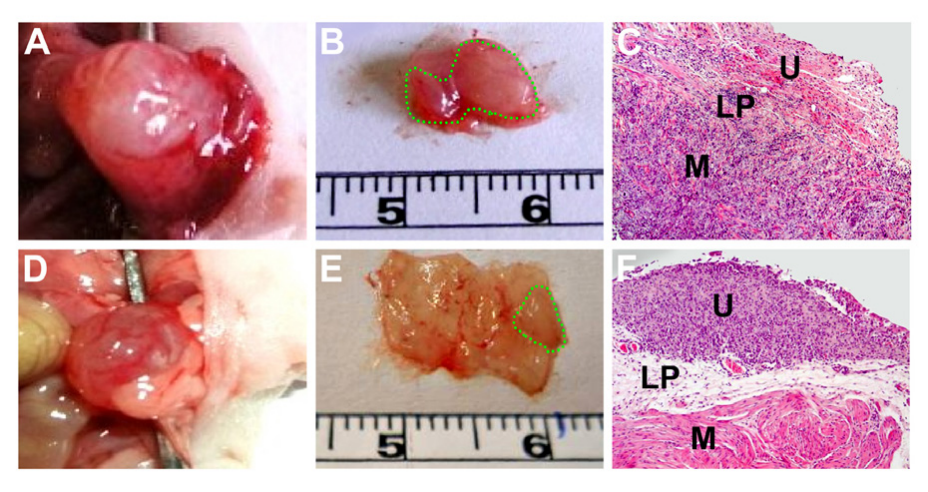
**Macroscopic and histopathological views of the orthotopic bladders treated with H19-DTA-P4-DTA**: Shown are macroscopic photographs of the whole orthotopic bladders treated with H19-Luc-P4-Luc (A), or with H19-DTA-P4-DTA (D). The bladders of both of the groups were excised, and the area of the malignant tissue of each bladder is indicated (by grin line) for the H19-Luc-P4-Luc (B) and H19-DTA-P4-DTA (E). Histopathological microscopic view (H&E × 10 is shown for H19-Luc-P4-Luc treated bladder (C), or with H19-DTA-P4-DTA treated bladder (F) and the tumor areas are indicated (by green line), ('U', urothelium,'LP', lamina propria, 'M', muscle).

Tumor area of each bladder was macroscopically determined, using the ImagePro Plus software for measurement and analysis of the tumor area. The average size of the H19-DTA-P4-DTA treated tumors at the end of the experiment was 86% smaller (Figure 8A) than that of the H19-Luc-P4-Luc treated ones (6.37 ± 2.1 mm^2 ^and 44.6 ±8.5 mm^2 ^respectively) (P < 0.001). As shown in Figure 9B, the group treated with the reporter vector showed usually more than one large lesion, with different grades of invasion. In contrast, only small tumors were detected in the H19-DTA-P4-DTA treated bladders (Figure 9E).

Inhibition of tumor growth was also reflected in bladders weight (Figure 8B). The mean bladder weight of H19-DTA-P4-DTA treated mice was 40 ± 9 mg compared to 120 ± 20 mg in the control group. The mean bladder weight of the healthy mice was 30 ± 3 mg (P < 0.001).

### Expression of DTA and Luc RNA in mouse orthotopic treated bladder tumors

**Figure 10:**
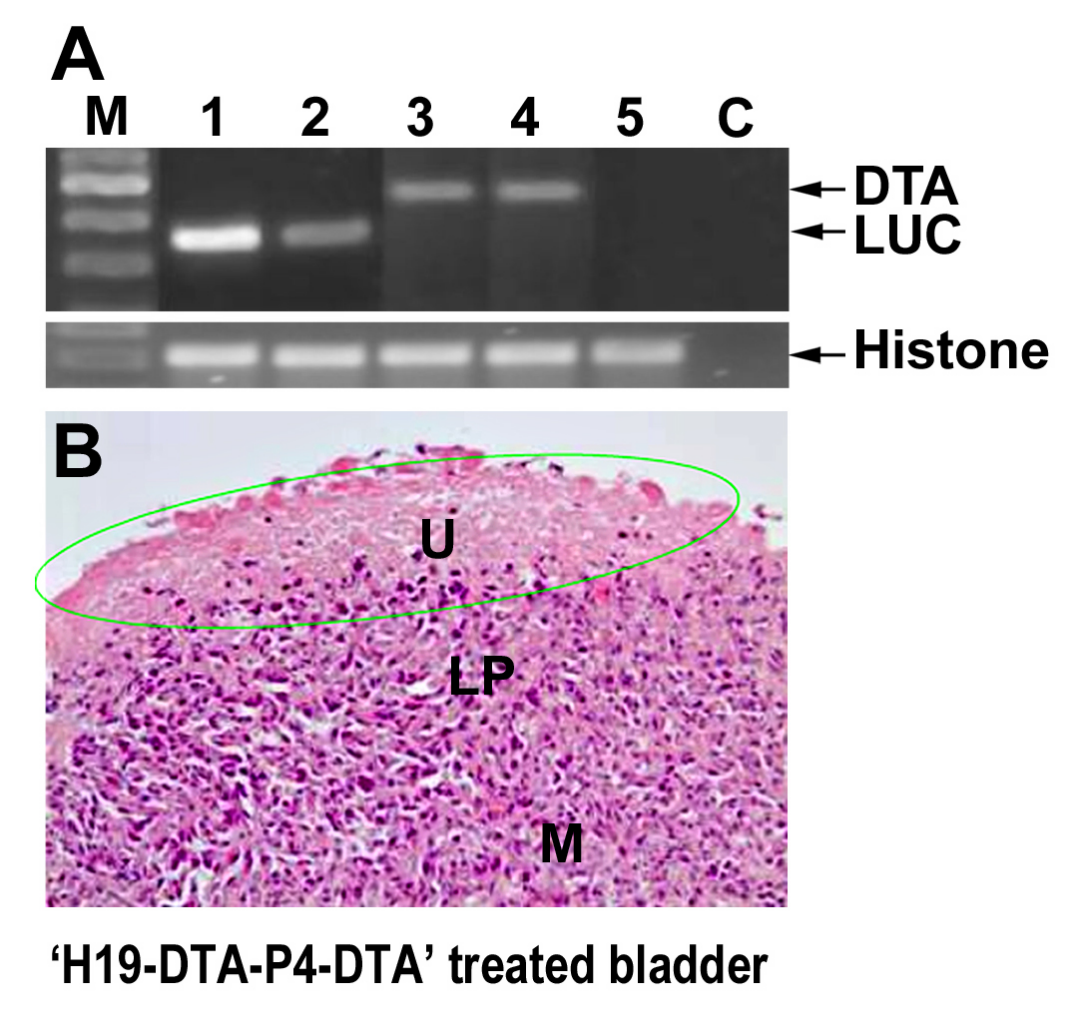
**Detection of DTA and Luc transcripts in orthotopic bladder tumors**: Mice with heterotopic bladder tumors were intravesically treated twice in 3 days interval, and were sacrificed 4 days after the last treatment. Tumors were excised and frozen immediately and 400 ng RNA (extracted from the tumors) was used for determination of luciferase and DTA by RT-PCR reaction. **A)**. tumors treated with H19-Luc-P4-Luc (lanes 1-2), or with H19-DTA-P4-DTA (lanes 3-4). Lane 5: untreated orthotopic bladder tumor, 'C': negative control for PCR, 'M': 100 bp DNA ladder. The sizes of the PCR products are 468 bp and 328 bp, for DTA and Luc respectively. The lower panel shows the histone 3.3 internal control. Necrosis of orthotopic bladder tumor treated with H19-DTA-P4-DTA (H&E × 20) is shown (**B**) and the necrotic area is indicated (by green line). ('U', urothelium, 'LP', lamina propria, 'M', muscle).

At the end of the experiment, bladders were excised and total RNA was extracted from each tumor. RNA samples from the treated tumors were analyzed by RT-PCR for DTA and for luciferase mRNA expression. Figure 10A (lanes 1-2) shows high luciferase expression after treatment with the H19-Luc-P4-Luc reporter vector.

The PCR revealed DTA mRNA expression in H19-DTA-P4-DTA treated tumors (lanes 3-4) but not in the luciferase treated tumors (lanes 1-2). This indicates that the tumors were efficiently transfected by the H19-DTA-P4-DTA vectors and that the human H19 and IGF2-P4 promoters were activated and DTA was produced.

Necrosis in H19-DTA-P4-DTA treated bladder, as a result of the diphtheria toxin activity, is shown in figure 10B

### In vitro enhanced activity of the double promoter H19-DTA-P4-DTA construct compared to combination of the single promoter constructs

**Figure 11:**
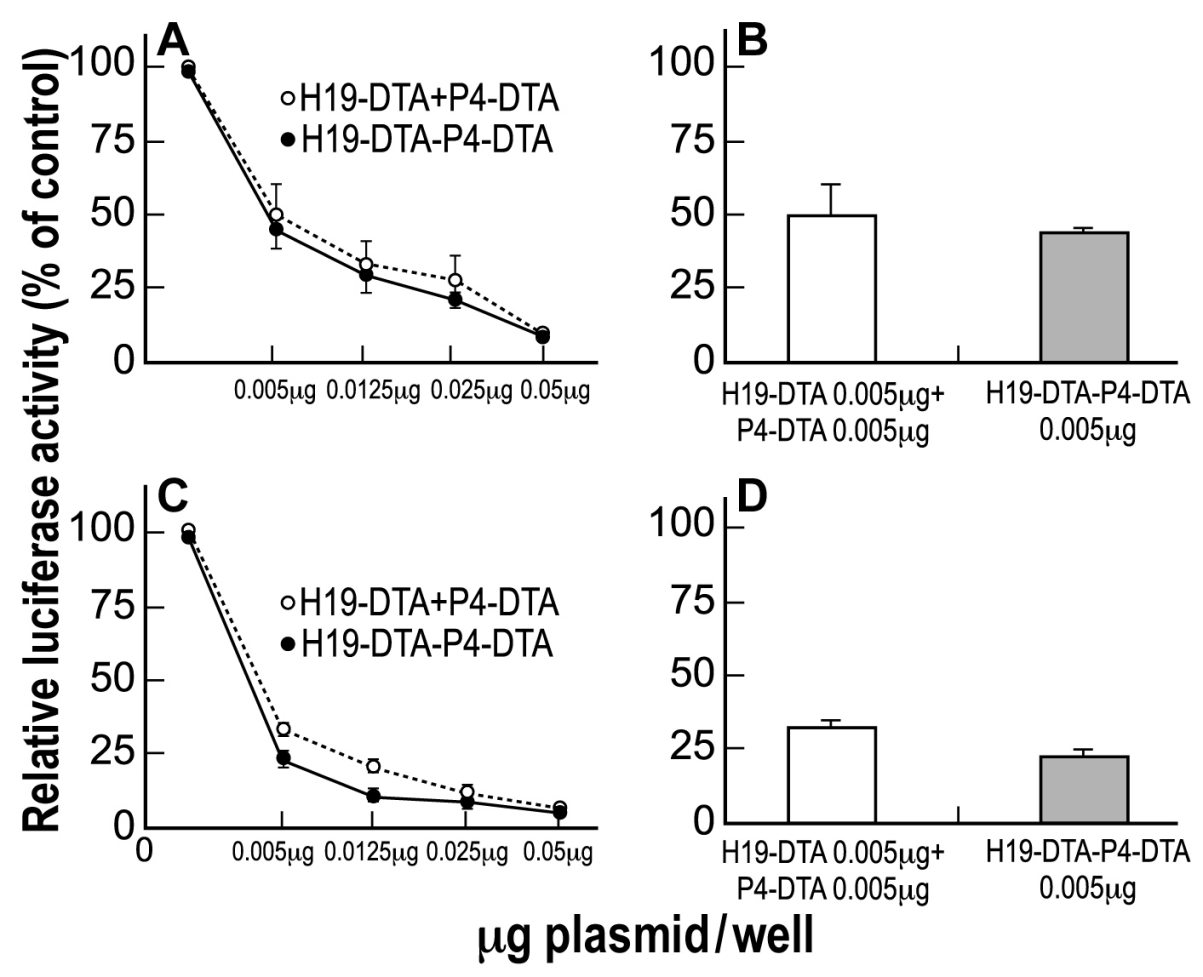
**Enhanced activity of H19-DTA-P4-DTA in human bladder carcinoma cell lines**: The protein synthesis inhibition activity of the H19-DTA-P4-DTA vector in T24P (A-B) and HT-1376 (C-D) cells was measured as a reduction of LucSV40 activity, and was compared to the combination activity of H19-DTA + P4-DTA. Cells were cotransfected with 2 μg of LucSV40, and with the indicated concentrations of the DTA expressing vectors or LucSV40 alone. Transfection experiments were stopped after 48 hours and luciferase activity was assessed. The decrease in LucSV40 activity was determined by comparison to the same cell type transfected with LucSV40 alone as a measure for cytotoxicity. Enhanced effect of H19-DTA-P4-DTA vector at the lowest plasmid transfected concentration (0.005 μg compared to 0.005 μg + 0.005 μg of the combination transfection of both vectors H19-DTA + P4-DTA) is indicated (B, D).

The presence of an enhanced anti-cancer activity of the double promoter construct H19-DTA-P4-DTA was tested in the human bladder cancer cell lines T24P and HT-1376. T24P and HT-1376 cells were co-transfected with 2 μg of LucSV40 and either (a) the concentrations indicated (Figure 11) of single-promoter constructs H19-DTA + P4-DTA in combination, or (b) the same amount of H19-DTA-P4-DTA as for one of the single-promoter constructs. The total amount of DNA co-transfected in samples receiving both single promoter constructs was therefore twice than the cells transfected with H19-DTA-P4-DTA. Luciferase activity was determined and compared to that of cells transfected with LucSV40 alone. The double-promoter construct H19-DTA-P4-DTA exhibited enhanced efficiency in lysing the cancer cell lines, relative to the combined activity of both single promoter constructs (H19-DTA + P4-DTA), in T24P cells (Figure 11A-B). Very similar results were obtained in HT-1376 cells (Figure 11C-D).

### In vivo additive activity of the double promoter construct compared to combination of the single promoter constructs

The presence of an additive tumor growth inhibition activity of the double promoter construct H19-DTA-P4-DTA was tested *in vivo *in a nude mice heterotopic bladder cancer model (described hereinabove). The therapeutic potency of the vector was tested by 3 intratumoral injections, at two-day intervals, of 25 μg of H19-DTA-P4-DTA or of the control vector (H19-Luc-P4-Luc), into each heterotopic bladder tumor. Tumor size was determined and *in vivo *fold increase of the tumor size was calculated at the end of each treatment.

To test whether the *in vivo *tumor growth inhibition activity of H19-DTA-P4-DTA was augmented-than-additive, an additional group of T24P tumor-containing mice was treated with three injections of 25 μg each of single-promoter constructs H19-DTA + P4-DTA in combination. The total amount of DNA co-transfected administered was therefore twice (50 μg) than the H19-DTA-P4-DTA group.

**Figure 12:**
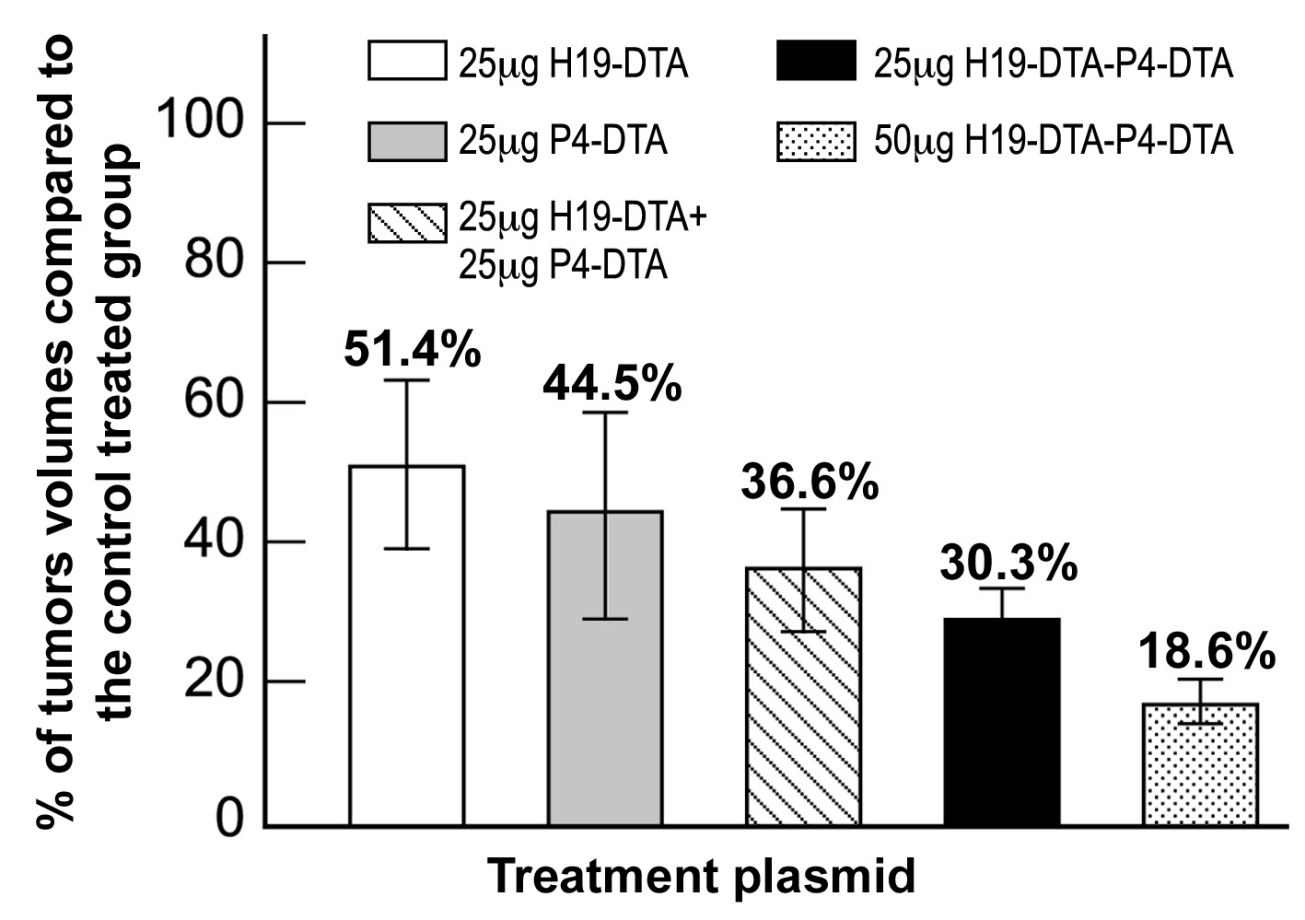
**Augmented-than-additive activity of H19-DTA-P4-DTA in heterotopic bladder tumors, induced by T24P cells**. The inhibition of heterotopic bladder tumor growth, induced by T24P cells is indicated by the fold increase of each DTA mice treated group compared to the control Luc treated mice. Shown are tumors treated with: 25 μg of H19-DTA, 25 μg of P4-DTA, 25 μg of H19-DTA + 25 μg of P4-DTA 25 μg of H19-DTA-P4-DTA and 50 μg of H19-DTA-P4-DTA.

As can be seen in Figure 12, tumor development in mice receiving both H19-DTA and P4-DTA plasmids was inhibited by 63.4% (P = 0.001) compared to combined H19-Luc + P4-Luc treated mice. However, an enhanced effect was observed in mice treated with the double-promoter construct H19-DTA-P4-DTA, wherein tumor development was inhibited by nearly 70% (P = 0.005) compared to mice treated with the control plasmid H19-Luc-P4-Luc. Figure 12 summarizes all T24P heterotopic bladder cancer model results. H19-DTA-P4-DTA clearly exhibits enhanced activity compared to each of the single promoter plasmids alone and also superior to their combined activity. As can be seen in Figure 12, mice intratumorally treated with higher dose as 50 μg of the double-promoter construct H19-DTA-P4-DTA (same total amount of the combined single promoter plasmids), showed enhanced inhibition of more than 80%.

Thus, the H19-DTA-P4-DTA vector exhibits augmented-than-additive *in vivo *tumor growth inhibition activity, compared to the combined activity of both single-promoter constructs (H19-DTA and P4-DTA).

## Discussion

The present work shows the successful use of a double promoter expressing vector, carrying on a single construct two separate DNA sequences expressing the diphtheria toxin A-fragment (DTA), from two different regulatory sequences, selected from the cancer-specific promoters H19 and IGF2-P4. This construct was used to transfect and to eradicate tumor cells in culture (*in vitro*) or tumors developed in animal models (*in vivo*) of bladder carcinoma.

Cancer is a multigene and multi-factorial disease. The last decade has seen the emergence of numerous multigene expression profiles that aim to outdo traditional predictive and prognostic factors (reviewed by [30]). However, targeted therapies such as Herceptin and Avastin, are targeting one specific protein. Further personalized and targeted therapies should be considered, targeting more than one target (protein or a gene). Accordingly, several chemotherapies nowadays are administrated as cocktails, in combination with radiotherapy (reviewed by [31]) and in combination with targeted agents (reviewed by [32]).

Therefore, we applied an innovative approach using on a single construct more than one specific marker gene which are differentially expressed in tumor cells, for targeted cancer therapy.

IGF2 and H19 are reciprocally imprinted and are highly expressed in a broad spectrum of tumors, but rarely in normal adult tissues [7,33]. Using a single promoter (e.g. an H19 promoter or an IGF2-P4 promoter) alone for expression of a cytotoxic gene presents several unresolved problems. For one, not every tumor cell of a given type of cancer is positive for expression via the H19 promoter or the IGF2-P4 promoter sequences.

Thus, such therapy could fail in a sizable proportion of patients, even without accounting for tumor mutagenesis. Determination of responsiveness to such constructs would involve the costly and difficult step of genotyping individual tumors.

Tumors are known to exhibit significant genomic instability and heterogeneity. Thus, even individuals with an H19-expressing tumor, for example, may contain some cancer cells that have downregulated or abrogated H19 expression via mutation. Therefore, expressing the cytotoxic gene from a single promoter in such patients may result in temporary and partial tumor regression that will rapidly be reversed when the cells containing these mutations survive and rapidly multiply.

Therefore the use of double promoter expressing vectors is highly novel. Tumor cells can express high levels of H19 and IGF2, or only one of those genes. That way, majority of the tumor cells could efficiently express the diphtheria toxin.

This novel approach, create a new family of plasmids regulated by two regulatory sequences, which in their natural genome position are both proximately located and are reciprocally imprinted. This is a novel biology concept, which mimics the unique biology reciprocity relations phenomenon of IGF2 and H19.

Once introduced into target tissue, the plasmid vectors have several advantages over viral vectors (reviewed by [34,35]): (1) the plasmids have no potential to be infectious; (2) they possess levels of expression per cell that are equivalent to some viral vectors that persist as extra-chromosomal elements; (3) the lack of immunogenicity, thus allowing for repeated treatments; (4) plasmids transfect mainly dividing cells, with contrast to most viral vectors that, except for retroviral vectors, which transfect both dividing and non dividing cells; and finally (5) the long-term stability, safety and the lack of need special treatments or storage requirement of the plasmid vectors.

In this study, the therapeutic potential of the vectors was tested in TCC of the bladder. The bladder has long been thought to be an ideal target for DNA based therapy because it is easily accessible by catheter and is largely a self-contained "bag-like" organ. While the protective glycosaminoglycan (GAG) that is present in the normal bladder mucosa interferes with the plasmid transfection [36], it is not present in the bladder tumor, allowing efficient transfection of principally the tumor urothelium. In the same way an orthotopic model can be designed, which the bladder can then be easily approached by catheter.

In cancer gene therapy, direct DNA injection is currently a reliable, reproducible, and simple technique for intratumoral gene transfer [37]. We transferred the plasmids into cell lines and into the target tissue of the animal models, as complex with the linear cationic polyethylenimine (jetPEI) as a transfection reagent. This method was chosen based on previous studies of our group showing relatively high levels of transfection efficiency, *in vitro*, *in vivo *and lately in TCC patients as part of a phase I/IIa bladder cancer clinical trial [25,27]. JetPEI condenses the DNA into positively charged particles capable of interacting with anionic proteoglycans at the cell surface and entering by endocytosis [38].

Subunit A of the diphtheria toxin (DTA), a highly potent poison, was chosen as an effector molecule. When only the cDNA coding for the A-fragment is expressed, the released DT-A toxin from the lysed cells will not be able to enter neighboring cells in the absence of the DT-B fragment [39]. This approach not only will insure high killing activity but will be of great advantage against any unintended toxicity to non-target normal cells. Moreover, introduction of DTA DNA sequence under the control of regulatory sequences of genes differentially expressed in tumors but not in adjacent non-tumor cells will selectively favor the specificity of the treatment.

Over plurality of cancer specific promoters, H19 and IGF2-P4 regulatory sequences were selected for targeting cancer cells. The H19 and IGF2-P4 regulatory sequences are expected to be good candidates for specifically inducing the expression of DTA in target tumor cells but not in cells of normal tissue. They are known to be differentially over-activated in various tumor types and to show no or minimum activity in the surrounding normal tissue [40,41]. This is in addition to the known autocrine/paracrine mode of IGF2 mitogen action in the development of a wide range of human malignancies. Accordingly, destruction of the H19 and IGF2 expressing tumor cells not only will eliminate part of the tumor but will also diminish the supply of mitogenic IGF2 to neighboring tumor and non-tumor cells and may lead to arrest of tumor growth and prevent following metastases process [42,43].

Based on previous results of our group demonstrating efficient treatment of TCC using either H19-DTA or IGF2-P4-DTA vector [25], it appeared that TCC tumors could be efficiently treated by each of these vectors. Based on this assumption we hypothesized that by using double promoter expression vector, which the expression of DTA is controlled by more than one regulatory sequence, a higher therapeutic potential is expected, if the tumor shows high specific expression from more than one of the above mentioned regulatory sequences (H19 or IGF2-P4).

In order to determine the applicability of this assumption, the first stage was to explore the expression level of each of the mentioned regulatory sequences and then compare it to the combined expression level (from the two regulatory sequences).

First, the overall expression of H19 and IGF2-P4 was analyzed by ISH and RT-PCR in 67 human TCC samples.

Taken together the PCR and ISH analyses results show (Table 1) that 62 out of 67 (92.5%) and 64 out of 67 (95.5%) positively expressed varying levels of IGF2-P4 and of H19, respectively.

Next, the quantitative expression was further analyzed by ISH and by qRT-PCR.

Out of 29 TCC samples detected by qRT-PCR, (Table 2), high levels of IGF2-P4 and H19 transcripts were found in 83% (24/29) and in 90% (26/29) of the tumor samples, respectively. Moreover, the total combined expression of both IGF2-P4 and H19 transcripts was detected at high expression levels in 100% (29/29) of the tumor samples.

Out of 28 TCC samples detected by ISH (Table 3), high levels of IGF2-P4 and H19 transcripts were found in 50% (14/28) and 75% (21/28) of the TCC samples respectively. When the overall combined expression analysis of the intensity and quantity of both transcripts H19 + IGF2-P4 was determined, then 100% (28/28) of the samples showed positive expression and 26 out of 28 TCC samples (96%) showed high expression.

Thus, both ISH and qRT-PCR detections confirmed that by analyzing the combined expression from two promoters, 100% of the samples show positive expression and nearly 100% show high expression.

These results clearly support the rationale of our hypothesis, which DTA could be extensively expressed from more than one specific regulatory sequence. Therefore, we further investigated the combination use of H19 and IGF2 regulatory sequences for driving toxin gene expression in therapeutic vectors for bladder cancer treatment.

The double promoter construct H19-DTA-P4-DTA exhibited far superior efficiency *in vitro *(Figure 3), in lysing human bladder carcinoma cell lines, relative to each of the single promoter constructs carrying either DTA DNA sequence alone (H19-DTA or P4-DTA).

Therefore we further evaluated the therapeutic potential of the double promoter toxin vector in heterotopic and orthotopic mouse models.

1. Heterotopic bladder cancer model was used to evaluate tumor growth inhibition of the double promoter vectors compared to that of the single promoter vectors. The advantages of this model are its rapidity, reproducibility, accessibility and visibility of tumors. When using immuno-deficient animal like the nude type mice, human cell lines can be employed and better simulation of human tumor is obtained. H19-DTA-P4-DTA exhibited superior ability to inhibit heterotopic tumor development by 70% (P < 0.001) compared to H19-DTA or P4-DTA activity (Figure 5).

Additional *Ex-vivo *measurements of tumors weight and volume, re-confirmed the difference between the H19-DTA-P4-DTA and control groups. The consistency of the results, by measuring of the *ex-vivo *tumors as well (Figure 6), eliminates any unrelated difference of the measurements (such as subcutaneous inflammation swelling due to necrosis reaction, etc.).

2. The disadvantage of the heterotopic model is the weak correlation in histology and clinical course between this model and the clinical disease. Therefore by inducing orthotopic TCC tumors in mice bladders, tumors resemble human bladder tumors by their histology, by the clinical course of TCC (local tumor growth, invasion, and metastatic activity), and by the ability to treat bladder tumors intravesically, the same way human bladders are clinically treated. Therefore we evaluated the feasibility of intravesical therapy of H19-DTA-P4-DTA, in nude mice orthotopic bladder cancer model. The average size of the H19-DTA-P4-DTA treated tumors was 86% smaller than that of the H19-Luc-P4-Luc treated ones (P < 0.001) (Figure 8A) and there was also significant difference in mean bladders weight (P < 0.001) (Figure 8B). Only small tumors were detected in the H19-DTA-P4-DTA treated bladders (Figure 9), compared to large lesions and with different grades of invasion in the group treated with the reporter vector. However the tumors were not completely destructed and it should be stressed that in patients with bladder cancer, the tumors are first surgically completely resected and the purpose of the following intravesical treatment is therefore to treat any possible remaining tumor cells and to prevent tumor recurrence.

The inhibition of tumor progression resulted exclusively from the toxic effect of the diphtheria toxin. This was confirmed by RT-PCR determining mRNA expression of DTA only in heterotopic tumors treated with DTA expressing vector (Figure 10A), and by performance of cellular necrosis in H19-DTA-P4-DTA treated tumors compared to the H19-Luc-P4-Luc treated and non-treated ones (Figure 10B).

In addition, all of the tested orthotopic tumor samples showed high expression of H19 and IGF2-P4 transcripts (Figure 7), thus it strongly prove the assumption that the orthotopic tumor cells activate the H19 and IGF2-P4 promoters and therefore drive the expression of DTA within the cells and in consequence triggering their necrosis.

Finally, we dealt with the question whether transfection of both single promoter vectors (expressing the diphtheria toxin) in combination, may exhibit better efficacy than transfection of the double promoter construct. The use of double promoter vectors was previously described [44] as a convenient tool for evaluation of the activity of a gene of interest by monitoring a reporter gene activity simultaneously expressed on the same construct. However an additive activity of the double promoter vector versus combination of two single promoter vectors was never demonstrated. Therefore the presence of an **additive **anti-cancer effect of the double promoter constructs H19-DTA-P4-DTA was tested *in vitro*, in human TCC cells and *in vivo*, in heterotopic bladder cancer mice.

*In vitro *enhanced activity of the double promoter vector H19-DTA-P4-DTA (Figure 11) was exhibited in T24P bladder cancer cells. A superior activity of the double promoter vector in lysing the cancer cell lines was exhibited, relative to the combined activity of both single promoter constructs (H19-DTA + IGF2-P4-DTA), in a dose response manner. It should be stressed that the total amount of DNA co-transfected in cells receiving both single promoter constructs was therefore twice than the cells transfected with the double promoter constructs.

Thus, H19-driven and IGF2-P4-driven DTA-encoding sequences presented on a single expression vector (H19-DTA-P4-DTA), exhibited enhanced protein synthesis inhibition activity, relative to expression vectors carrying either DTA sequence alone when tested against bladder cancer cells.

**Augmented-than-additive **activity of the double promoter vectors H19-DTA-P4-DTA (Figure 12) was further exhibited *in vivo*, in heterotopic tumors induced by T24P bladder cancer cell lines. Heterotopic tumors treated with combination of total amount of 50 μg of both single promoter H19-DTA and -P4-DTA constructs, were inhibited by 63% (P = 0.001) compared to combined H19-Luc + P4-Luc, control treated mice (Figure 12). However, an enhanced effect was observed in mice treated with only 25 μg of the double-promoter construct H19-DTA-P4-DTA, wherein tumor development was inhibited by 70% (P = 0.005) compared to the mice treated with the control plasmid H19-Luc-P4-Luc.

Tumors treated with higher dose as 50 μg of the double-promoter construct H19-DTA-P4-DTA (same total amount of the combined single promoter plasmids), showed enhanced inhibition of at least 80% (Figure 12). Thus, the H19-DTA-P4-DTA vector exhibited augmented-than-additive *in vivo *anti-cancer activity, compared to the combined activity of both single-promoter constructs (H19-DTA and P4-DTA.

## Conclusions

In this study double promoter expression vector were used, expressing DTA from two different regulatory sequences, H19 and IGF2-P4.

Several reasons support this strategy. First, IGF2-P4 and H19 are reciprocally imprinted and are exclusively expressed at high levels in cancer cells and not in normal cells. We demonstrated that combined expression from the two separate regulatory sequences, showed complementary expression profile, in which nearly 100% of tumor samples expressed high levels from at least one of the regulatory sequences. By that the DTA could be better expressed in larger number of cancer cells and therefore enhance the tumor inhibition activity.

Second, H19 and IGF2 play major role in tumor development. By selective killing of cancer cells, which express H19 and IGF2, the treated tumor cells as well as the neighboring tumor cells (as IGF2 mediate its effect in autocrine/paracrine manner) are at least partly deprived of their IGF2 supply. By that the targeted destruction of cancer cells expressing IGF2 or H19, companied by enhanced bystander effect, may lead to arrest of tumor growth and prevent following metastases process.

Overall, the double promoter vector, H19-DTA-P4-DTA, exhibited augmented-than-additive anti-cancer activity relative to single promoter expression vectors carrying either DTA sequence alone, when tested against bladder tumor cells.

As H19 and IGF2-P4 are expressed at very high levels in a broad spectrum of different cancers, therefore we propose a double promoter expression approach for targeted cancer therapy. According to this approach patients will be treated with specific double promoter expression toxin vector which are under the control of the IGF2-P4 and H19 regulatory sequences, differentially expressed in those cancers.

Moreover, our proposed treatment may be applied in combination with other cancer therapy methods, such as chemotherapy and radiology. This approach should be tested in appropriate animal models.

## List of abbreviations

**ATCC**: American type culture collection; **BCG**: Bacillus Calmet-Guerin; **DTA/DT-A**: Diphtheria toxin A chain; **H19-Luc-P4-Luc**: Reporter vector expressing each luciferase under the control of a different promoter: H19 or IGF2-P4; **H19-DTA-P4- DTA**: Therapeutic (double promoter) vector expressing each DTA under the control of a different promoter: H19 or IGF2-P4; **IGF2 - **Insulin like growth factor 2; **ISH**: In situ hybridization; **Luc**: Luciferase; **P4**: Human IGF2 P4 promoter; **H19-Luc**: Reporter vector expressing the luciferase under the control of human H19 promoter; **P4-Luc**: Reporter vector expressing the luciferase under the control of human IGF2 P4 promoter; **H19-DTA**: Therapeutic (single promoter) vector expressing the DTA under the control of H19 promoter; **P4-DTA**: Therapeutic (single promoter) vector expressing the DTA under the control of IGF2 P4 promoter; **PCR**: Polymerase chain reaction; **PEI**: Polyethylenimine; **TCC**: Transitional cell carcinoma; **qRT-PCR**: quantitative real-time polymerase chain reaction

## Competing interests

The authors declare that they have no competing interests.

## Authors' contributions

DA - conducted the study and conceived of the study, participated in design, coordination, data interpretation, performed the statistical analysis, and drafted the manuscript. AH - conceived of the study, participated in design, interpretation of data and critically revised the manuscript. All authors read and approved the final manuscript.
